# Validation of the Danish version of the Carer Experience Scale in family caregivers of people with dementia

**DOI:** 10.1186/s12955-026-02477-0

**Published:** 2026-01-29

**Authors:** Emma Kjær Pedersen, Laila Øksnebjerg, Gunhild Waldemar, Janet Janbek, Ann Nielsen, T. Rune Nielsen

**Affiliations:** 1 Danish Dementia Research Centre, Department of Neurology, Copenhagen University Hospital - Rigshospitalet, Copenhagen, Denmark; 2 Department of Psychology, University of Copenhagen, Copenhagen, Denmark; 3 Department of Clinical Medicine, University of Copenhagen, Copenhagen, Denmark

**Keywords:** CES, Caregiver outcomes, Family caregiver, Quality of life, Outcome measures, Dementia, Alzheimer’s disease

## Abstract

**Background:**

The Carer Experience Scale (CES) measures the caring experience, focusing on caregiver-related quality of life (QoL) rather than health-related QoL. We aimed to validate the Danish language version of CES in family caregivers of people with dementia in Denmark by assessing CES discriminative and convergent validity. Further, we aimed to establish the internal consistency and test-retest reliability of CES.

**Methods:**

A baseline questionnaire from the Danish DemTool intervention trial, including CES and five other measures of QoL and wellbeing was completed by 375 family caregivers of people with dementia. Discriminative validity was assessed by the ability of CES to discriminate between different caring contexts and levels of caregiver strain, as determined by Mann–Whitney *U* and Kruskal–Wallis *H* tests. Convergent validity was assessed by analyzing correlations using Spearman’s rank correlation coefficients between the CES index and domain scores, and the 5-level EQ-5D version (EQ-5D-5L), European Quality of life visual analog scale (EQ VAS), Neuropsychiatric Inventory Caregiver Distress Scale (NPI-D), World Health Organization Wellbeing Index (WHO-5), and the University of California, Los Angeles Three-Item Loneliness Scale (UCLA 3-item). Internal consistency was assessed using Cronbach’s α and test-retest reliability was assessed using intraclass correlation coefficient (ICC) between two different time points.

**Results:**

The CES effectively distinguished between different levels of caregiver strain. Further, the CES was not affected by different caregiver and care recipient characteristics. Supporting convergent validity, the CES index was moderately correlated with NPI-D (ρ=-0.42), WHO-5 (ρ = 0.44), and UCLA 3-item (ρ=-0.40). The CES index was weakly correlated with EQ-5D-5L (ρ = 0.27), and EQ-VAS (ρ = 0.26). The CES showed low internal consistency (α = 0.53), but high test-retest reliability (ICC = 0.76).

**Conclusion:**

The Danish version of CES demonstrated discriminative validity and acceptable psychometric properties, providing further evidence for its use in family caregivers of people with dementia.

**Supplementary Information:**

The online version contains supplementary material available at 10.1186/s12955-026-02477-0.

## Background

In Denmark, it is estimated that approximately 97,000 individuals aged 65 and older have dementia [1]. This number is expected to increase in the next decades due to the increasing number of older adults in the Danish population [2]. Dementia is a complex syndrome characterized by a gradual onset and a progressive decline in cognitive functions. It may also involve various behavioral and psychological symptoms, such as depressive mood and aggression [3]. These behavioral and psychological symptoms often lead to high levels of distress and anxiety for both individuals with dementia and their family caregivers [4, 5]. Family caregivers may be defined as individuals who provide unpaid and ongoing assistance to a family member with a chronic illness or disability, or to an older adult who is unable to manage independently [6]. People with dementia often need comprehensive support, depending on the stage of the disease, from family members [7, 8] and from healthcare professionals [9]. Providing informal care can be fulfilling, and some family caregivers report positive experiences when undertaking this role [10]. However, it can also be challenging, and evidence indicates that informal caregiving is associated with increased levels of stress and may affect the caregiver’s quality of life [11–13] and physical and mental health [14–17]. As the disease progresses, family caregivers will continuously need to adapt to new situations due to changes in interrelational roles and positions, which present additional challenges [18]. Therefore, it is essential to apply relevant measures to assess the quality of life (QoL) of family caregivers and to apply these as essential outcome measures when conducting psychosocial intervention studies.

The experiences and burden of family caregivers are difficult to capture using current health-related QoL outcome measures [19], such as the EuroQol group five-dimension questionnaire (EQ-5D) [20], as the attributes captured may not reflect caregivers’ concerns about relational and psychosocial aspects [21].

Health-related QoL is commonly understood as a multidimensional construct that reflects an individual’s perceived physical, psychological, and social wellbeing, and includes both objective aspects, e.g., functional ability, symptom burden, and subjective perceptions, e.g., satisfaction, meaning, and personal appraisal of health [22, 23]. However, many generic health-related QoL measures do not sufficiently account for the complex, relational nature of caregiving. Therefore, there is a need for establishing and validating new scales addressing the experience of providing daily care [24]. The Carer Experience Scale (CES) was developed as a generic measure of the experiences of family caregivers across different areas of chronic illness [25, 26]. In contrast to health-related QoL measures, CES focuses on the caring experience and caregiver-related QoL. CES was developed specifically to reflect the lived experiences of family caregivers across different domains. Several studies have supported the use of CES for assessing QoL of family caregivers of persons within several chronic illness areas, including dementia [27, 28]. To assess the validity of CES and situate it among existing outcome measures, we included well-established measures that capture related but distinct domains, such as generic health-related QoL, caregiver distress, wellbeing, and loneliness. These measures have also been used in previous CES validation studies, facilitating cross-study comparison and supporting broader validation of the scale [14, 29]. Although previous research on the CES has highlighted the need for further validation in specific clinical subgroups, including relating the CES to established measures of caregiver strain [27], CES has so far only been validated in samples of family caregivers of people within an unspecified group of chronic illness in Australia and the United Kingdom [14, 27–32].

### Aim

This study aimed to validate the Danish language version of CES in family caregivers of people with dementia in Denmark by assessing its discriminative and convergent validity. This study further aimed to establish the internal consistency and test-retest reliability of the CES.

## Methods

### Data collection and design

Data were derived from a baseline questionnaire from the Danish DemTool intervention trial. Briefly, DemTool was developed by the Danish Dementia Research Centre [33] and is a pragmatic psychosocial multicomponent intervention for people with dementia and their family caregivers, with the aim of supporting everyday life across various dementia diseases and dementia stages. DemTool comprises seven interventions, including individual counseling and group-based activities, delivered as lectures, group courses, and support groups. The DemTool intervention trial was conducted in people with dementia and their family caregivers across 30 Danish municipalities in 2020–2023. Data for the present validation study comprised baseline data from adult family caregivers of people with dementia participating in the DemTool intervention trial. All participants completed a sociodemographic and health questionnaire, the CES, and standardized measures of health-related quality of life, distress, wellbeing, and loneliness. Baseline data were collected 1–14 days before the family caregivers received their first intervention of DemTool. Additionally, retest data were collected for a subsample of 52 family caregivers in the treatment-as-usual group within 3 months (mean: 64 days, ± 10 days, range: 20–90 days) from baseline.

### Carer Experience Scale

CES is a preference-based measure of caregiving experience, focusing on six domains: activities outside caring, support from family and friends, assistance from government and other organizations, fulfillment from caring, control over caring, and getting on with the care recipient [25–27]. The CES consists of six items in total, with each item corresponding directly to one of these six domains. A high index score indicates less strain from the caregiver experience. CES is rated on a three-point Likert scale, where each attribute either relates to an amount or a frequency. As in previous studies, a total index score from 0 to 100 was calculated by summing the preference weights for each selected domain [28]. These preference weights were established through best-worst scaling in a sample of informal caregivers in the United Kingdom [27].

### Questionnaire preparation

The translation of the CES was inspired by established principles for cross-cultural adaptation of self-report instruments, described by Sousa and Rojjanasrirat [33]. The CES was first translated into Danish by a bilingual researcher familiar with questionnaire development and translation, and then independently back-translated by another bilingual researcher unfamiliar with the original scale, but familiar with scale development and translation. These two translations of the scale were compared with the original version by the two researchers, discrepancies were discussed and resolved, and a pre-final version of CES was decided on.

**Fig. 1:**
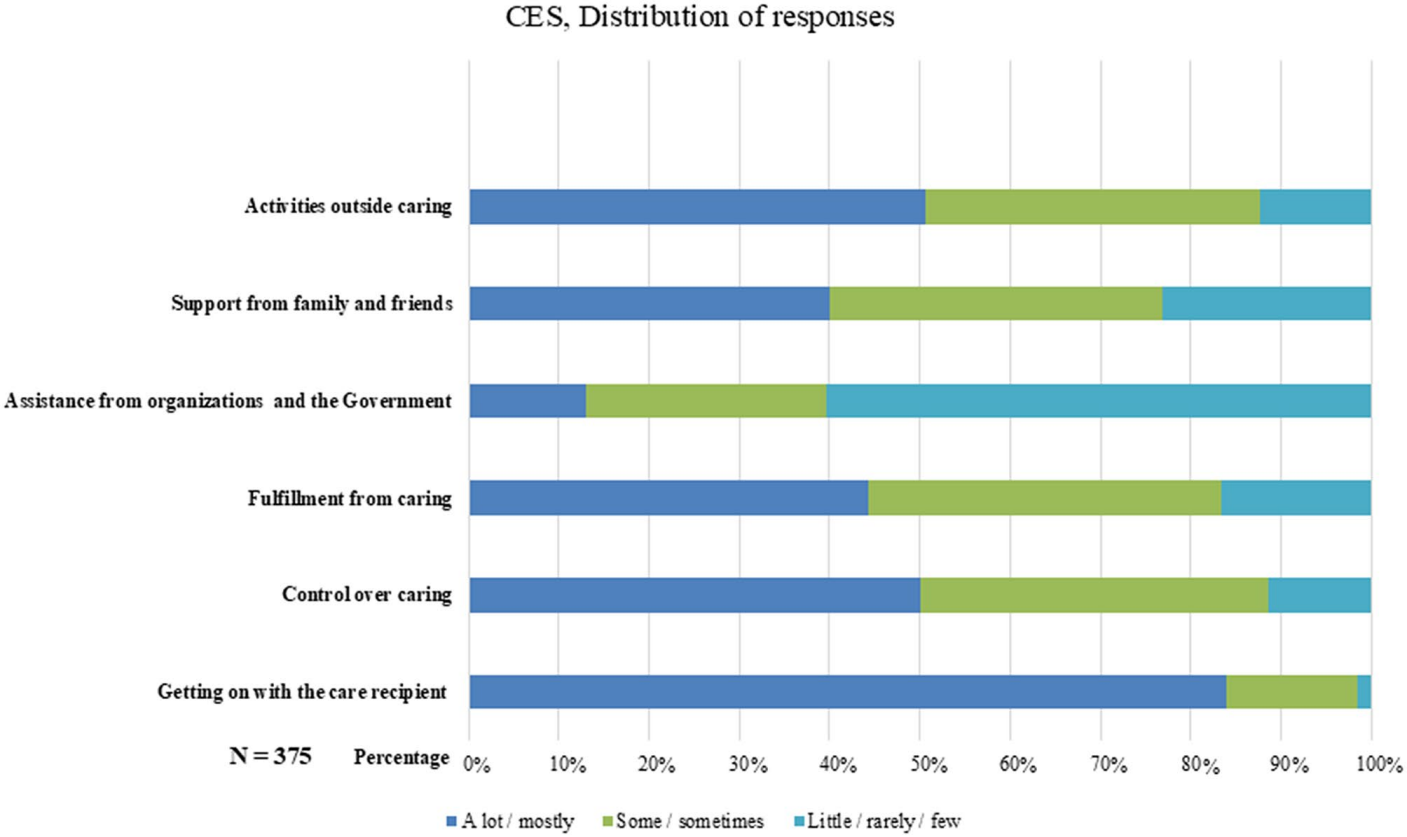
Distribution of responses of the Carer Experience Scale

Following this, we pilot tested the pre-final Danish version through cognitive interviews with four family caregivers. While no changes were made to the CES, participants reported question three: *“Indicate which statement that best describes your current caring situation regarding assistance from government and organizations (help from public, private or voluntary groups in terms of benefits, respite, and practical information)”* to be confusing. A flow chart of the translation and validation process can be found in the supplementary See Fig. 1S.

### Procedures and other assessments

According to individual preferences, participants completed the baseline questionnaire online (SurveyXact) (79.4%), in a printed paper version (14.7%), or through telephone interviews (5.9%). The baseline questionnaire included CES as well as characteristics of the caregiver and care recipient, and standardized questionnaires measuring caregiver strain: The 5-level EQ-5D version (EQ-5D-5L) [20, 34, 35], European Quality of life Visual Analog Scale (EQ VAS), which is not a separate measure, but rather a stand-alone component of the EQ-5D-5L [36]. Further, standardized questionnaires were the Neuropsychiatric Inventory Caregiver Distress Scale (NPI-D) [37]; World Health Organization Wellbeing Index (WHO-5) [38, 39]; and University of California, Los Angeles Three-Item Loneliness Scale (UCLA 3-item) [40, 41]. See Table 1 for a description of CES and measures of caregiver strain.

### Aspects of validity

Discriminative validity was assessed by the ability of the CES index to reflect expected differences in informal caregiver situation groups. Convergent validity was assessed by analyzing the correlation between the CES index and domain scores, and caregiver strain measures. Before conducting the analysis, the following a priori hypotheses were stated:

1. We hypothesized weak or no correlations between CES and health-related quality of life measures (EQ-5D-5L, EQ VAS), as CES captures caregiving experience rather than general health status.
2. We hypothesized that the type of relationship to the person with dementia (e.g., spouse vs. other) would differentiate CES scores, as these caregiver groups often have different caregiving experiences.
3. We hypothesized a moderate negative correlation with NPI-D, as caregiver distress may influence the caregiving experience, though not strongly, given that CES is not a symptom-specific measure.

Internal consistency was established using Cronbach’s α for the CES index, and test-retest reliability was assessed using intraclass correlation coefficient (ICC) between two different time points.

**Table 1:** Detailed description of the included rating scales

Measure	Construct	Questions/Domains	Scoring procedure	Scale range
CES [25–27]	Caregiver experience	1) Activities 2) Support 3) Assistance 4) Fulfillment 5) Control 6) Getting on	The answer from each domain is converted to a preference weight and summed	0 to 100 Higher index: less strain from the caregiver experience.
EQ-5D-5 L [20, 34, 35]	Health-related quality of life	1) Mobility 2) Self-care 3) Usual activities 4) Pain/discomfort 5) Anxiety/depression	The answer from each domain is converted into utility scores. When a state deviates from ‘perfect health’ a preferred weight is deducted from the starting point	1 to -0.757 Higher score: better health-related quality of life
EQ VAS [36]	Health-related quality of life	Self-reported general health	Read from the graph	0 to 100 Higher score: better health-related quality of life
NPI-D [37]	Distress	1) Delusions 2) Hallucinations 3) Dysphoria/depression 4) Agitation/aggression 5) Anxiety 6) Apathy/indifference 7) Euphoria/elation 8) Irritability/lability 9) Disinhibition 10) Aberrant motor behaviors 11) Sleep 12) Appetite changes	Sum distress score from each domain. When a symptom is not present the distress level equals 0	0 to 60 Higher score: higher distress
WHO-5 [38, 39]	Mental wellbeing	1) Positive mood and wellbeing 2) Inner calm 3) Energy and vitality 4) Sleep quality and restoration 5) Meaningfulness and engagement	Sum score for each domain and multiplied by 4	0-100 Higher score: better wellbeing
UCLA 3-item [40, 41]	Loneliness	1) Relational connectedness 2) Social connectedness 3) Self-perceived isolation	Sum score for each domain	3–9 Higher score: higher loneliness

*CES*: Carer Experience Scale. *EQ-5D-5L*: The 5-level EQ-5D version. *EQ VAS*: European Quality of life Visual Analog Scale. *NPI-D*: Neuropsychiatric Inventory Caregiver Distress Scale. *WHO-5*: World Health Organization Wellbeing Index. *UCLA 3-item*: University of California, Los Angeles Three-Item Loneliness Scale

### Statistical analyses

Data management and statistical analyses were conducted using SAS Enterprise Guide Version 8 software. A p-value < 0.050 (two-tailed) was considered significant. Educational level was categorized according to ISCED 2011. Levels 1–2 (primary and lower secondary education) were grouped as low education, level 3–4 (upper secondary and short-cycle tertiary education) as medium education, and levels 5–7 (bachelor’s, master’s, or equivalent) as high education [42, 43]. All statistical tests were performed using nonparametric methods, as the assumptions of normally distributed residuals within each group and equal variances were not satisfied. Discriminative validity was assessed using the nonparametric Mann–Whitney *U* and Kruskal–Wallis *H* tests. Effect sizes were calculated and interpreted as follows: Cohen’s r for Mann–Whitney U with 0.11–0.30 indicating small, 0.31–0.50 moderate, and ≥ 0.51 indicating large effects; Eta squared for Kruskal–Wallis H with 0.01–0.059 indicating small, 0.06–0.139 moderate, and ≥ 0.14 indicating large effects. To facilitate the interpretation of the discriminative validity of the CES in relation to caregiver strain, all outcome measures were grouped into low, medium, and high categories. As no established clinical cut-offs exist for the EQ-5D-5L, EQ VAS, or NPI-D, the thresholds for these measures were derived empirically from the distribution of scores in our sample and used solely for descriptive grouping. For the WHO-5, the lower cut-off followed the validated threshold for possible depression (≤ 50) [38], whereas the upper category was defined based on the score distribution. For the UCLA 3-item, categorization followed existing literature, distinguishing between “not lonely” (3–5) and “lonely” (6–9) [44]. 

Convergent validity was assessed using Spearman’s rank correlation coefficients between the CES index and domain scores, and EQ-5D-5L, EQ VAS, NPI-D, WHO-5, and UCLA 3-item scores. Correlations were interpreted according to Cohen’s guidelines, with 0.11–0.30 indicating weak, 0.31–0.50 moderate, and ≥ 0.51 indicating strong correlations [45]. Internal consistency was established using Cronbach’s α for the CES index with a threshold of 0.70. Cronbach’s α was interpreted as: 0.70–0.80 indicating acceptable, 0.80–0.90 good, and ≥ 0.90 indicating excellent internal consistency [46]. Test-retest reliability of the CES was assessed using ICC. ICC values were interpreted as: < 0.50 indicating poor, 0.51–0.70 moderate, 0.71–0.90 good, and ≥ 0.90 indicating excellent reliability [47]. Effect sizes of moderate magnitude or above were considered acceptable indicators of validity and reliability [48].

### Power calculation

Power calculations were performed based on the original validation study of the CES [27]. The number of family caregivers needed to identify a difference between different levels of caregiver strain, defined as a difference of 3.5 points on the CES index (α = 5%, β = 20%, expected SD: 5.0), was 256.

## Results

Of 390 family caregivers in the DemTool intervention trial, 15 were excluded due to incomplete CES data. Of those, 11 failed to respond to question three (regarding assistance from government and organizations). Thus, the final sample consisted of 375 family caregivers of people with dementia (intervention, *n* = 294; treatment as usual, *n* = 81).

### Participant characteristics

Participant characteristics are described in Table 2. The majority of family caregivers were women (75%) aged ≥ 70 years (52%), who cared for a community-dwelling (86%) spouse, partner, or cohabitant (70%). The care recipients were mostly men (58%), aged 70–80 years (45%), and 34% had received a dementia diagnosis ≤ 12 months before baseline. According to WHO-5, 40% of family caregivers reported experiencing poor mental wellbeing, 36% reported feelings of loneliness according to UCLA 3-item, 11% had a low health-related quality of life according to EQ-5D-5L, and 2% had a high level of distress according to NPI-D.

**Table 2:** Characteristics of sample

Caring context	*n*(%)	CES index, Mean (SD)	*p*-value
**Caregiver characteristic**			
Sex (*n* = 375)			
Male Female	95 (25) 280 (75)	70.1 (16.8) 68.8 (18.6)	*p* = 0.734
**Age (*n* = 363**,** mean: 68.2; SD: 11.2)**			
≤ 55 > 55 and < 70 ≥ 70	49 (14) 125 (34) 189 (52)	68.2 (18.6) 68.8 (19.1) 69.2 (17.3)	*p* = 0.982
**Highest attained educational level (** ***n*** ** = 374)**			
Low Medium High	57 (15) 156 (42) 161 (43)	73.0 (18.0) 69.1 (18.9) 67.8 (17.3)	*p* = 0.117
**Relation to the care-recipient (** ***n*** ** = 375)**			
Spouse/cohabitant/partner Other	264 (70) 111 (30)	67.9 (18.4) 72.0 (17.0)	***p*** ** = 0.043**
**Care recipients**			
**Sex (** ***n*** ** = 375)**			
Male Female	217 (58) 158 (42)	68.4 (18.5) 70.1 (17.6)	*p* = 0.394
**Age (** ***n*** ** = 371, mean: 77.8; SD: 7.5)**			
≤ 70 > 70 and < 80 ≥ 80	54 (15) 167 (45) 150 (40)	67.0 (21.2) 68.9 (17.7) 70.2 (17.4)	*p* = 0.647
**Time since diagnosis (** ***n*** ** = 369)**			
≤ 12 months 1–2 years ≥ 3 years	154 (42) 106 (29) 109 (29)	71.2 (17.9) 69.9 (15.5) 64.9 (20.4)	***p*** ** = 0.031**
**Residence (** ***n*** ** = 374)**			
Community-dwelling Residential care	322 (86) 52 (14)	68.4 (17.9) 73.0 (19.0)	*p* = 0.058
**Caregiver strain**			
**EQ-5D-5 L (** ***n*** ** = 374)**			
Low (≤ 0.65) Medium (> 0.65 and < 0.81) High ≥ 0.81)	40 (11) 43 (11) 291 (78)	60.8 (17.9) 62.3 (21.7) 71.3 (17.0)	***p*** ** < 0.001**
**EQ VAS (** ***n*** ** = 372)**			
Low (≤ 65) Medium (> 65 and < 81) High (≥ 81)	105 (28) 102 (28) 165 (44)	62.5 (20.5) 69.1 (15.8) 73.2 (16.7)	***p*** ** < 0.001**
**NPI-D† (** ***n*** ** = 375)**			
Low distress (≤ 12) Medium distress (> 12 and < 36) High distress (≥ 36)	189 (51) 177 (47) 9 (2)	75.7 (14.7) 63.6 (18.3) 39.6 (15.5)	***p*** ** < 0.001**
**WHO-5 (** ***n*** ** = 375)**			
Low (≤ 50) Medium (> 50 and < 75) High (≥ 75)	148 (40) 143 (38) 84 (22)	59.8 (18.7) 72.9 (16.3) 78.94 (11.2)	***p*** ** < 0.001**
**UCLA 3-item† (** ***n*** * = 375)*			
Not lonely (≥ 3 and ≤ 5) Lonely (≥ 6 and ≤ 9)	239 (64) 136 (36)	73.9 (16.4) 60.8 (18.0)	***p*** ** < 0.001**

*EQ-5D-5L*: The 5-level EQ-5D version. *EQ VAS*: European Quality of life visual analog scale. *NPI-D*: Neuropsychiatric Inventory Caregiver Distress Scale. *WHO-5*: World Health Organization Wellbeing Index. *UCLA 3-item*: University of California, Los Angeles Three-Item Loneliness Scale. Education: grouped according to ISCED levels: ISCED 1–2 = low, ISCED 3–4 = medium, ISCED 5–7 = high. †: Higher scores represent more negative effects; **Bold** results indicate a *p* < 0.050

### Discriminative validity

The CES index significantly distinguished between groups with different times since diagnosis (H: 6.94, *p* = 0.031, η^2^: 0.02); and relation to care recipient (U: 2.02, *p* = 0.043, r: 0.01) (see Table 2). Also, CES indexes distinguished between groups with different levels of caregiver strain measured with: EQ-5D-5 L (H: 16.94, *p* < 0.001, η^2^: 0.05); EQ VAS (H: 20.66, *p* < 0.001, η^2^: 0.06); NPI-D (H: 60.00, *p* < 0.001, η^2^: 0.16); WHO-5 (H: 69.43, *p* < 0.001, η^2^: 0.19); UCLA 3-item (U: -6.89, *p* < 0.001, r: 0.13) (see Table 2). No significant differences were found in other group comparisons (see Table 2).

### Convergent validity

The CES index was moderately correlated with NPI-D, WHO-5, and UCLA 3-item, and weakly correlated with EQ-5D-5L and EQ VAS (see Table 3). The CES domains were most strongly correlated with the domain ‘activities outside caring’. None of the measures were significantly correlated with the domain ‘assistance from government and organizations’ (see Table 3).

**Table 3:** Convergent validity of the Carer Experience Scale

Measure	CES index	CES domains					
		Activities outside of caring	Support from family and friends	Assistance from government and organizations	Fulfillment from caring	Control over caring	Getting on with the care-recipient
EQ-5D-5 L	**0.27**	**0.37**	**0.11**	-0.04	**0.18**	**0.14**	**0.19**
EQ VAS	**0.26**	**0.29**	**0.14**	-0.05	**0.18**	**0.19**	**0.18**
NPI-D	**-0.42**	**-0.44**	**-0.17**	-0.06	**-0.33**	**-0.26**	**-0.39**
WHO-5	**0.44**	**0.47**	**0.26**	0.03	**0.29**	**0.23**	**0.32**
UCLA 3-item	**-0.40**	**-0.45**	**-0.28**	-0.04	**-0.23**	**-0.17**	**-0.19**

*CES*: Carer Experience Scale. *EQ-5D-5L*: The 5-level EQ-5D version. *EQ VAS*: European Quality of life Visual Analog Scale. *NPI-D*: Neuropsychiatric Inventory Caregiver Distress Scale. *WHO-5*: World Health Organization Wellbeing Index. *UCLA 3-item*: University of California, Los Angeles Three-Item Loneliness Scale. Correlations interpreted as: 0.11–0.30 indicating weak, 0.31–0.50 moderate, and ≥ 0.51 strong correlations. **Bold** results indicate a *p* < 0.050

### Reliability

Cronbach’s alpha for the CES index was 0.53, indicating poor internal consistency. ICC was 0.76, indicating good test-retest reliability.

### Distribution of responses

The mean CES index was 69.1, SD: 18.1. Overall, family caregivers reported positive experiences in their caregiving roles, except for the level of assistance from government and organizations (see Fig. 1).

## Discussion

This study is the first to validate the CES in a sample of family caregivers of people with dementia in Denmark. The CES effectively distinguished between different levels of caregiver strain and was not affected by different caregiver and care recipient characteristics. Moderate correlations between CES and NPI-D, WHO-5, and UCLA 3-item supports its convergent validity. While CES showed poor internal consistency, it had high test-retest reliability.

Our findings add to earlier validation studies of the CES. The literature indicates weak but significant correlations of the discriminative validity of CES on caregiver strain, related to hours of care and the number of caregiver tasks. These studies were conducted in Australia [28] and the United Kingdom [27], looking at samples of family caregivers of people within an unspecified group of chronic illnesses. The Australian sample included family caregivers of people with dementia. Their findings align with ours on characteristics of caregiver strain. We found a weak correlation between the CES index and the EQ-5D-5L, consistent with existing literature in a sample of family caregivers in the United Kingdom caring for adults with a chronic condition, including dementia [29]. The weak correlation likely reflects differences in which aspects of caring the two rating scales aim to assess. In dementia caregiving, the strain is often more psychosocial than physical [49], which EQ-5D-5L may not fully capture. In addition, our results of the discriminative validity of CES supported our second a priori hypothesis. Spouses reported significantly lower CES scores than other caregiver groups (*p* = 0.043), which could indicate that spouses experience more emotionally demanding caregiving roles compared to other groups of family caregivers.

In our analysis of CES convergent validity, we found a moderate correlation between the CES index and UCLA 3-item, which was also reported in a sample of family caregivers in England, primarily caring for people with physical or intellectual disabilities or mental health conditions [14]. The negative correlation between the UCLA 3-item, NPI-D, and the CES is rational, as a higher index of CES reflects greater positive experiences. Consequently, it can be inferred that when family caregivers report a high CES index, feelings of distress and loneliness are likely not present. The negative and moderate correlation between CES and NPI-D confirms our third hypothesis. Further, we found nonsignificant results for question three (assistance from government and organizations), suggesting that this question does not contribute with any additional information. We also noted potential floor effects within this question (see Fig. 1), which may hinder detecting an association. We did not include a measure capturing a construct entirely unrelated to caregiving experiences, which limited our ability to assess divergent validity formally. However, comparisons with conceptually distinct measures, like health-related QoL assessed with EQ-5D-5L and EQ VAS, indicate expected weaker associations, which also confirms our first hypothesis.

As for the CES internal consistency, our analysis of family caregivers of people with dementia aligns with existing literature in a sample of Australian family caregivers of people with a chronic condition, including dementia, as a Cronbach’s alpha below the recommended 0.70 threshold was reported [28]. This was expected, as CES captures the overall caregiving experience through six distinct domains of caregiver experiences using one item per domain. Consequently, we should not expect CES to have high internal consistency. A lower Cronbach’s alpha, therefore, reflects the heterogeneous nature of the caregiving experience rather than a concern regarding the measurement properties of the scale.

CES showed a high intraclass correlation, indicating good test-retest reliability, which aligns with existing literature in a sample of Australian family caregivers of people with a chronic condition, including dementia [28]. The results of CES internal consistency and test-retest reliability support the use of CES for family caregivers of people with dementia in the Danish context. Cognitive interviews conducted during the pilot study revealed that family caregivers found question three (assistance from government and organizations) difficult to comprehend, as its meaning was unclear to them. This aligns with the literature, as one study indicated low feasibility of this specific question in a sample of family caregivers of older adults in the United Kingdom [32].

A strength of this study is the large sample size of family caregivers of people with dementia, rather than samples of family caregivers caring for people spanning multiple disease categories, as seen in previous studies [14, 27–32]. Another strength is that the CES was validated alongside standardized caregiver strain measures, such as the EQ-5D-5L, WHO-5, and NPI-D. This study also has some limitations. As it sampled a homogeneous group of family caregivers from the DemTool trial, there was an overrepresentation of older adults and more women, so the perspectives of younger and male caregivers were underrepresented. However, this seems to reflect a general issue, as research indicates that women commonly assume caregiving roles [50], making our population representative. Unlike other studies [27–29] we did not collect data on the number of years spent on caregiving, whether the caregiver was the primary caregiver, and time spent on caregiver tasks. Therefore, it is challenging to make direct comparisons of caregiver strain across studies. Lastly, we did not collect information on caregivers’ own health conditions (e.g., self-reported chronic diseases). Such data could have provided additional context for interpreting CES scores. Despite these limitations, our results generally align with the existing literature on caregivers to people with chronic disorders, providing further evidence for the validity of the CES [14, 27–31].

The results of this study indicate that the Danish version of CES is a valid and reliable measure for assessing the experiences of family caregivers of people with dementia. However, question three, which addresses assistance from the government and organizations, did not perform well. Therefore, it may be considered to omit or rephrase question three for the CES in the Danish context. Despite this concern, CES seems to be a promising outcome measure for assessing the broader effects of psychosocial interventions on caregivers’ quality of life beyond health-related factors, as CES encompasses a wide range of aspects of caregiving. CES is particularly relevant when evaluating interventions that address the caregiver and care-recipient relationship. In future studies, it will be relevant to include CES as an outcome measure in psychosocial intervention trials. Further validation is also warranted in caregiver groups with different care situations and caregiving dynamics, for example, caregivers of people with young-onset dementia, adult children caring for a parent, or caregivers of individuals with rare dementia diagnoses such as frontotemporal dementia. Additionally, exploring the use of the CES among ethnically diverse populations is important, as cultural norms and expectations may significantly influence the caregiving experience. These groups may face distinct challenges that could affect how the CES captures their lived experiences.

## Supplementary Information

Below is the link to the electronic supplementary material.

Supplementary Material

## Data Availability

The datasets analyzed during the current study are not publicly available, as we do not have approval for open access from the participants.

## Abbreviations

CES: Carer Experience Scale
EQ-5D-5L: The 5-level EQ-5D version
EQ VAS: European Quality of life visual analog scale
H: Kruskal-Wallis Test statistic H
ICC: Intraclass Correlation Coefficient
NPI-D: Neuropsychiatric Inventory Caregiver Distress Scale
QoL: Quality of life
SD: Standard deviation
U: Mann-Whitney Test statistic U
UCLA 3-item: University of California, Los Angeles Three-Item Loneliness Scale
WHO-5: World Health Organization Wellbeing Index
