# Supplementary material for: Validation of the Danish version of the Carer Experience Scale in family caregivers of people with dementia
Source: Health Qual Life Outcomes. 2026 Jan 29;24:14. doi: 10.1186/s12955-026-02477-0 (PMC12857010; doi:10.1186/s12955-026-02477-0)
Supplement: Supplementary file 1 — Supplementary Material [file 12955_2026_2477_MOESM1_ESM.docx]

**Supplementary material**

**Figure 1s - Flow chart of the translation and validation process of the Danish version of CES**

**
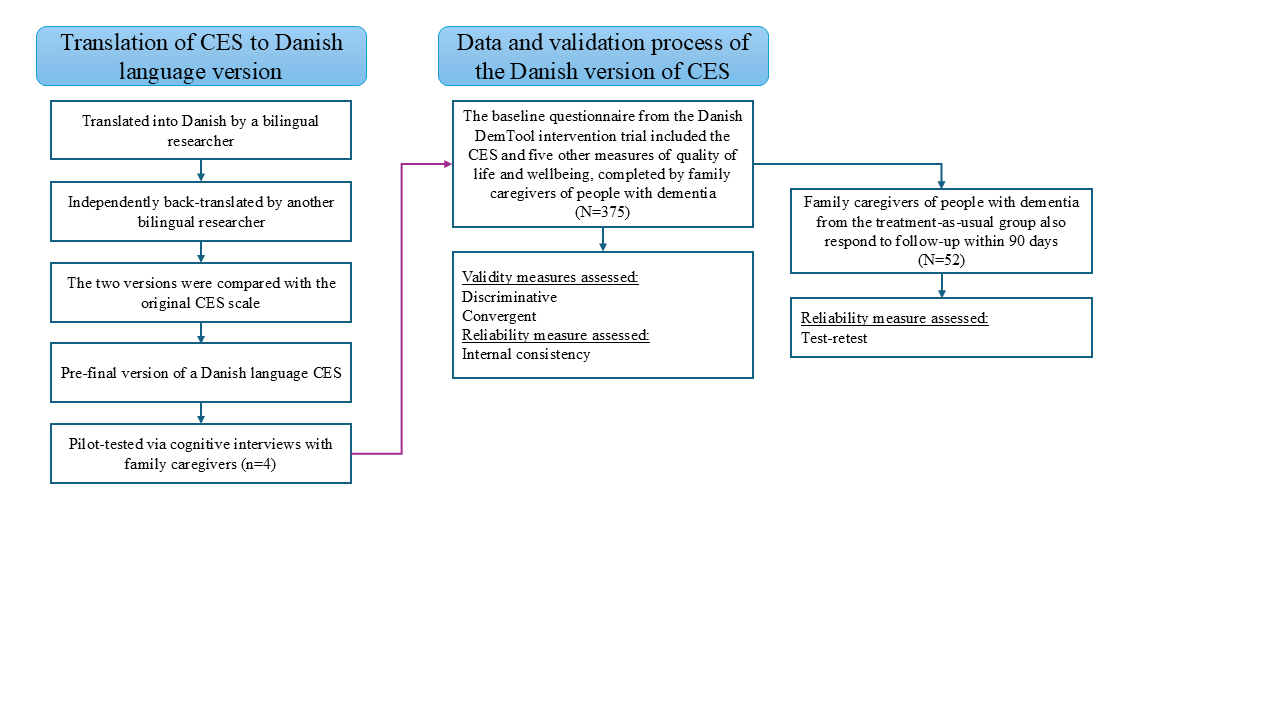
**
